# Cell Adhesion, Morphology, and Metabolism Variation via Acoustic Exposure within Microfluidic Cell Handling Systems

**DOI:** 10.1002/advs.201902326

**Published:** 2019-10-30

**Authors:** Citsabehsan Devendran, James Carthew, Jessica E. Frith, Adrian Neild

**Affiliations:** ^1^ Laboratory for Micro Systems Department of Mechanical and Aerospace Engineering Monash University Clayton VIC 3800 Australia; ^2^ Department of Materials Science and Engineering Monash University Clayton VIC 3800 Australia

**Keywords:** acoustofluidics, cell handling, cell viability, microfluidics, surface acoustic waves

## Abstract

Acoustic fields are capable of manipulating biological samples contained within the enclosed and highly controlled environment of a microfluidic chip in a versatile manner. The use of acoustic streaming to alter fluid flows and radiation forces to control cell locations has important clinical and life science applications. While there have been significant advances in the fundamental implementation of these acoustic mechanisms, there is a considerable lack of understanding of the associated biological effects on cells. Typically a single, simple viability assay is used to demonstrate a high proportion of living cells. However, the findings of this study demonstrate that acoustic exposure can inhibit cell attachment, decrease cell spreading, and most intriguingly increase cellular metabolic activity, all without any impact upon viability rates. This has important implications by showing that mortality studies alone are inadequate for the assessment of biocompatibility, but further demonstrates that physical manipulation of cells can also be used to influence their biological activity.

## Graphical abstract

Adoption of acoustofluidic cell handling within clinical and life science applications warrants a thorough understanding of the associated biological impact. Reported findings reveal biological impact on cell adhesion, morphology, and an increased metabolism as a result of specific acoustic exposure conditions while mortality rates remain unaffected, highlighting the inadequacy of single cell viability assays for these systems.

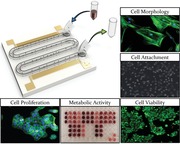

## 1 Introduction

The manipulation of biological matter has become widespread in microfluidics.^1^, ^2^, ^3^ The benefits of operating at a reduced size scale have enabled highly efficient techniques for selective patterning and manipulating cells.^4^, ^5^ Such approaches have been used for a wide range of tasks, including single cell analysis,^1^, ^3^, ^6^, ^7^ tissue engineering,^8^, ^9^, ^10^, ^11^, ^12^ studying cell–cell interaction and signaling,^2^, ^13^, ^14^ sorting,^5^, ^15^, ^16^, ^17^ and drug screening.^18^ As the applications of this technology are focused within the clinical and life sciences, a thorough understanding of the associated biological impact imposed by these manipulation techniques is necessary.

The desire to manipulate suspended matter within microfluidic systems has inspired a range of techniques both passive^5^, ^19^, ^20^ and active.^21^, ^22^ Passive approaches rely heavily on the geometry of the microfluidic channels and inertial forces. Flow profiles are altered by the introduction of sudden expansions and contractions, weirs and pillars to impede and divert the flow into desired streamlines. This reliance on the resultant flow profile restricts the flexibility of the chip, being single task specific. In contrast, active methods are significantly more robust, capable of on‐demand actuation and offer the ability to change functionality ad‐hoc, leading to an increased selectivity. To this end, a range of active methods have been developed using magnetic,^23^, ^24^ optical,^25^, ^26^ electrical^16^, ^27^ and acoustic^28^, ^29^, ^30^ excitation.

Acoustofluidics is the use of acoustic forces to manipulate suspended matter within microfluidics,^31^, ^32^ and has the advantage of uniquely combining ease of on‐chip integration and simple, yet dextrous establishment of force fields in a noncontact manner.^29^, ^33^, ^34^ As a direct result, it has been extensively used to capture,^33^, ^35^ pattern,^36^, ^37^, ^38^ and sort particles,^15^, ^34^, ^39^ cell sonoporation,^40^ synthesize nanomaterials,^41^ as well as to mix fluids.^42^, ^43^ Although acoustofluidics has been widely accepted as a biocompatible technique, substantiated with cell viability studies;^44^, ^45^, ^46^, ^47^, ^48^ there have been no extensive viability studies at elevated frequencies (30–600 MHz). Indeed, typically studies are corroborated with a single viability method, most commonly live/dead staining,^6^, ^49^, ^50^, ^51^ or trypan blue exclusion^52^, ^53^ and in some instances proliferation studies (MTT).^36^ In contrast to these singular approaches, in passive microfluidic systems in which cells are predominantly subjected to shear forces arising from the flow field, a wide range of multifaceted cell viability studies^54^ have been conducted showing, for example, shear‐dependent regulation of the von Willebrand factor of human umbilical vein endothelial cells^55^ and the potential for circulating tumor cell apoptosis at high shear levels. 56

This lack of biological knowledge may result in potential unrecognized adverse effects (i.e., “false positives”), or underexploited beneficial effects, based on the current biocompatible analysis methods, hampering direct translation of these technologies within clinical and life science applications. To address this inadequacy and to understand the associated biological effects of high frequency ultrasound, we probe a range of cell lines, using a suite of techniques chosen to determine postacoustic exposure effects on proliferation, membrane permeability, metabolic rate, cell attachment, and morphology. Four distinct cell lines were used, with known variation in cell stiffness and from two species (human and mouse). They were HaCaT (human keratinocytes), L929 (mouse fibroblast), MSCs (mesenchymal stromal cells, human bone marrow‐derived primary cells), and MG63 (mouse osteosarcoma). The selected cells represent not only species and source tissue variability but also vast differences in predicted cell stiffness values, ranging from 0.8 kPa (MSCs^57^, ^58^) to 10 kPa (HaCaT^59^) to better understand the impact of acoustic exposure on cell heterogeneity. Experiments we have performed show no significant differences in nuclear morphology and proliferation rates across all cell types and conditions studied. However, we did observe significant variations in cell attachment, spreading and metabolic activity, over a range of shear stresses induced by flow and acoustic exposures, differences that would otherwise have remained undetected due to the consistently high viability percentage observed in standard live/dead assay data. Investigation of the effect of acoustic excitation powers, flow rates, and channel dimensions on cell behavior is found to have a consistent increase in metabolic activity across cell lines in response to acoustic exposure.

## 2 System Principles

A major reason to use high frequency (30–600 MHz) ultrasound in a microfluidic system is that the wavelength is in the same order as that of a typical cell (i.e., 2–50 µm). This is a prerequisite for patterning of single cells,^6^ but has also been shown to provide the possibility of high sensitivity sorting,^15^, ^33^, ^60^ patterning using either standing waves^61^, ^62^ or traveling waves^32^, ^63^ and fluid mixing protocols.^43^ Excitation can be achieved using surface acoustic waves, these waves are substrate bound until they encounter a fluid body whereupon energy is efficiently coupled. An alternating current is applied, at the operational frequency (48.5 MHz used here; 80 µm surface acoustic waves (SAW) wavelength, λ_SAW_), to a set of opposing interdigitated transducers (IDTs) patterned on a piezoelectric substrate (128° Y‐cut X propagating Lithium Niobate used; LiNbO_3_), resulting in a standing wave that couples directly as a pressure field within the fluid medium containing cells.^6^ Typically, two main forcing mechanisms are present, namely acoustic radiation forces and acoustic streaming induced drag forces. The former acts on the suspended matter as a result of its interaction with the incident and scattered acoustic waves, whereas, the latter drags the particle in steady state fluid flows driven by Reynolds stresses.^31^

The relative significance of these two forces can be altered based on the excitation parameters such as the frequency, as well as the channel geometry, particle and fluid properties.^64^ Here, the streaming effects are minimal in comparison to the radiation force based on the frequency and channel dimensions used.

**Figure 1:**
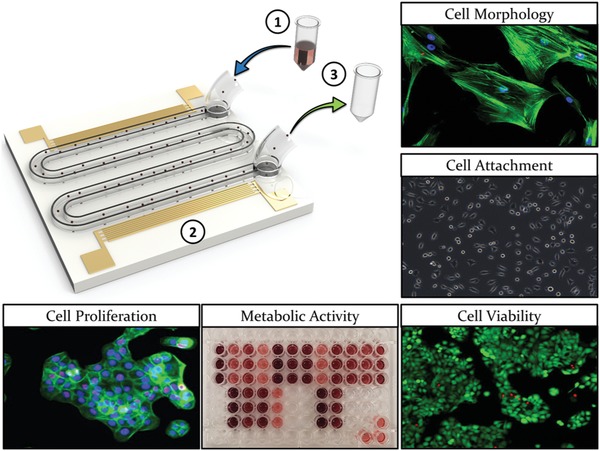
Design concept depicting the serpentine channel and IDT design. Distinct cell types are 1) fed (indicated by the blue arrow) into the system at a constant flow rate, 2) exposed to the ultrasound, and 3) retrieved (indicated by the green arrow) prior to seeding into tissue culture plastic (TCP) for various assessment techniques (see Figure S1 in the Supporting Information for the process diagram).

The microchannel was designed as a serpentine, as shown in **Figure** **1** (the experimental device has 11 turns as opposed to the 3 turns shown). The curves of the serpentine were sufficiently large that the shear stress profile is largely unchanged from the straight part of the channel (see Figure S2, Supporting Information). It was necessary to have a continuously flowing system to ensure that sufficient numbers of cells could be passed through the sound field, while the serpentine design facilitated prolonged exposure times. Both the shear stress and exposure times were varied by using combinations of two different channel heights (25 and 50 µm) and two flow rates (5 and 10 µL min^−1^). The microfluidic chip was temperature controlled using a Belektronig Benchtop Temperature controller to avoid heating of the cells during acoustic excitation.

## 3 Results

### 3.1 Cell Morphology and Substrate Attachment

**Figure 2:**
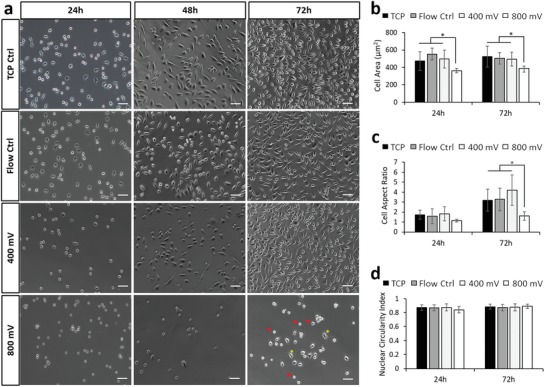
Acoustic exposure resulted in decreased cell attachment speeds for L929 cells, while presenting minimal impact on nuclear morphology. a) Phase contrast images of L929 cells 24, 48, and 72 h postexposure across each experimental condition. Arrows depict cells displaying inhibited substrate attachment, while asterisks depict normally spread cells. Quantification of observed b) cell area, c) cell aspect ratio, and d) nuclear circularity for L929 cells across 24 and 72 h postexposure. Data are presented as mean ± SD from triplicate samples, *n* = 9 (> 600 cells per time point) for each condition tested, with data analyzed using one‐way ANOVA supplemented with Tukey post hoc testing. Scale bar, 50 µm. Statistically different samples are denoted by *p < 0.05.

As an initial, simple readout of cell behavior in response to acoustic exposure, the adhesion and morphology of cells retrieved from the device was determined. Cells subjected to acoustic exposure were compared to i) cells that were simply plated into standard tissue culture wells (tissue culture plastic (TCP) control) and ii) cells that were passed through the device in the absence of acoustic stimulation (flow control). These controls provided a benchmark to account for any effects of detaching and manipulating the cells or exposing them to fluid flow within the device. For the L929 cell line (**Figure** **2**a), no differences in cell adhesion were observed in response the flow control or at the lower level of 400 mV acoustic excitation. However, cell adhesion and spreading were affected when an excitation amplitude of 800 mV (indicated by the arrows in Figure 2a) was used. Under these conditions, the cells remained circular with no spreading—this is a particularly striking alteration as no signs of recovery were observed even after 72 h as demonstrated by quantification of cell area and cell aspect ratio as shown in Figure 2b,c, respectively. Interestingly, no differences were observed in the nuclear circularity (Figure 2d). It is well established that nuclear abnormalities are associated with a diseased state (most prominent in cancer; nuclear blebbing and enlarged nuclei), thus, assessment of nuclear circularity was conducted to rule out this adverse scenario.

**Figure 3:**
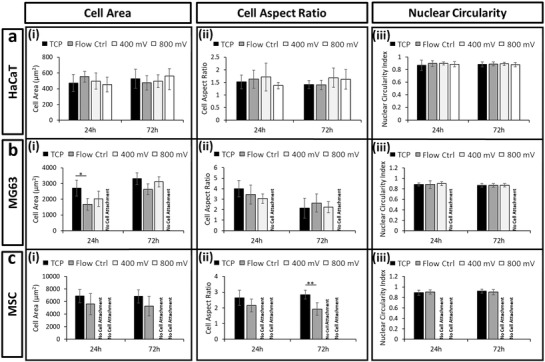
Acoustic exposure resulted in limited changes to cell phenotypes. Quantification of i) cell area, ii) cell aspect ratio, and iii) nuclear circularity for a) HaCaT, b) MG63, and c) MSC cells across 24 and 72 h postexposure. Labels of no cell attachment denote scenarios in which cells could not adhere to the growth substrate postexposure and thus could not be assessed. Data are presented as mean ± SD from triplicate samples (>600 cells per time point), with data analyzed using one‐way ANOVA with Tukey post hoc testing. Statistically different samples are denoted by *p < 0.05, **p < 0.005.

The data for the other three cell types, MSCs, MG63, and HaCaT, are shown in **Figure** **3** (images shown in Figure S3, Supporting Information). For the HaCaT cells (Figure 3a), no significant variation in morphology was observed across cell area, aspect ratio and nuclear circularity, for all conditions and time points. The higher resistance to morphological change as a result of an external stressor may be attributed to the abundant expression of keratin within these cells,^65^ which makes for a relatively stable structure. For the MG63 cells (Figure 3b) and MSCs (Figure 3c), no significant change was observed for the flow control samples over 24 h, while the highest acoustic power resulted in a complete inability for cells to attach to the substrate. For the MSCs, which are known to be extremely mechanosensitive and thus relatively more susceptible to external stressors, attachment did not occur even at the lower acoustic power level (see Figure 3c).

### 3.2 Cell Viability and Metabolic Activity

**Figure 4:**
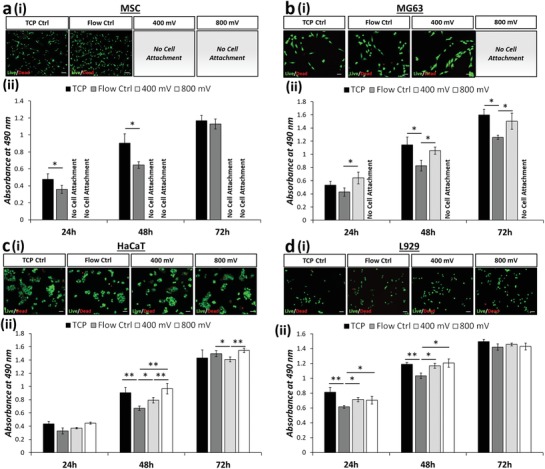
Variability is evident between viability assays designed to target membrane permeability and metabolic activity. Live/dead (green/red) fluorescence staining i) 24 h postexposure (see Table S1 in the Supporting Information for tabulated live cell percentage) and ii) formazan absorbance (MTS assay; metabolic activity) are presented for a) MSCs, b) MG63, c) HaCaT, and d) L929 cells. MTS assay data are presented as mean ± SD from triplicate samples (*n* = 9) for 24, 48, and 72 h time points postexposure, with data analyzed using one‐way ANOVA with Tukey post hoc testing. Labels of no cell attachment denote scenarios in which cells could not adhere to the growth substrate postexposure and thus could not be assessed. Scale bar, 50 µm. Statistically different samples are denoted by *p < 0.05, **p < 0.005.

Cell viability is commonly measured either using live/dead staining as a simple way to discriminate viable cells or assays that use cellular metabolism as a surrogate marker, such as MTS (a novel tetrazolium compound [3‐(4,5‐dimethylthiazol‐2‐yl)‐5‐(3‐carboxymethoxyphenyl)‐2‐(4‐sulfophenyl)‐2H‐tetrazolium, inner salt; (MTS^(a)^]). We therefore performed both of these assay types to examine the impact of acoustic stimulation upon the different cell populations. Importantly, although the live/dead data showed very little variation across treatment and cell type (**Figure** **4**a‐i–d‐i and Table S1, Supporting Information), the metabolic data revealed several significant effects. First, we saw that simply by passing the cells through the microfluidic chip, there was a dip in metabolic activity (Figure 4a‐ii–d‐ii) which lasted for up to 72 h, compared to the TCP control. However, this was mitigated when acoustic actuation was applied and the metabolic readings were comparable to the TCP control at the highest power level. Two possible hypotheses are that 1) the acoustic field decreases the effects of shear induced by the fluid flow—this could occur due to acoustophoretic particle migration toward the center line of the channel,^15^ and hence away from the high shear regions at the periphery of the channel, or 2) the acoustic fields are stimulating an increase in metabolic activity irrespective of shear. This could occur either directly or by indirectly acting upon currently undefined cellular mechanotransduction signaling pathways. Although the observed metabolic activity trend was similar across all the data obtained, the data set is not full, as the reduced adhesion of MSCs and MG63s under acoustic stimulation mean that data could not be collected for these conditions.

### 3.3 Cell Proliferation

**Figure 5:**
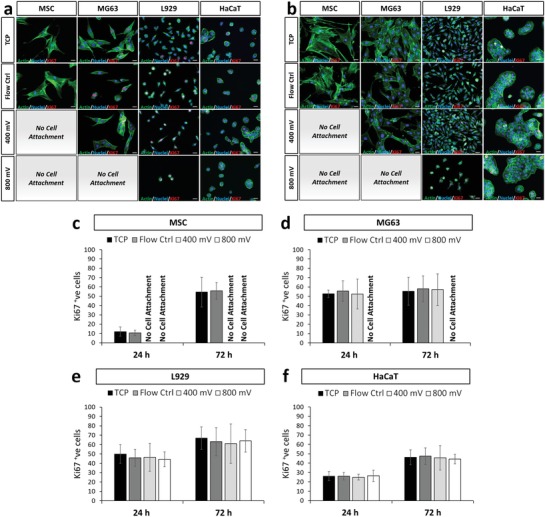
Postexposure assessment revealed no significant changes in cell proliferation rates for each cell type tested. Fluorescence observations of each cell type a) 24 and b) 72 h postexposure. Staining depicts f‐actin (green), nuclei (blue), and Ki67 (red). Labels of no cell attachment denote scenarios in which cells could not adhere to the growth substrate postexposure and thus could not be assessed. Scale bar, 20 µm. Quantification of Ki67 positive cells are presented for c) MSCs, d) MG63, e) L929, and f) HaCaT cells across 24 and 72 h postexposure. Data are presented as mean ± SD from triplicate samples, *n* = 9 (>600 cells per time point) for each condition, with data analyzed using one‐way ANOVA supplemented with Tukey post hoc testing.

Although MTS (and similar) assays can be used over time as an indicator of cell proliferation, they are not strictly markers of cell proliferation and rely on the assumption of equal metabolic activity in all cells and under all conditions. For this reason, we also assessed a more direct marker of cell proliferative capacity—Ki67 staining. Ki67 is expressed during all active phases of the cell cycle (G1, S, G2, and mitosis), but is absent in resting (quiescent) cells (G0). Overall expression of Ki67 increases during cell progression through S phase of the cell cycle. Therefore we used staining for Ki67 to determine the number of actively dividing cells at a particular snapshot in time (i.e., at 24 h (**Figure** **5**a) and at 72 h (Figure 5b)); Ki67 marker shown in red).

By comparing the proportion of Ki67‐positive (Ki67^+^) nuclei, it was clear that the proliferation rate of different cell types naturally varies. This can be expected as these cells are inherently different in terms of functionality and tissues are well known to have different turnover rates.^66^ For example, 24 h postexposure, only 10% of MSCs were shown to express detectable levels of Ki67 in comparison to MG63 cells in which 55% of cells were Ki67^+^. However, within a single cell type no significant difference in Ki67 activity, and hence proliferation rate, was detected (Figure 5c–f) while maintaining a consistent cell number across all time points considered (see Figure S4, Supporting Information). This confirms that cell proliferation was not affected by flow through the device or acoustic stimulation. Furthermore, these data collectively indicate that the variations in MTS assay data, as reported in Figure 4a‐ii–d‐ii, are due to specific variations in metabolic activity rather than proliferation rate.

### 3.4 Probing Shear Effects via the Modification of Channel Design and Flow Rates

Two hypotheses could explain the data presented in Figure 4: 1) acoustophoretic migration away from the channel periphery mitigates the effects of shear induced by flow and 2) the acoustic excitation directly causes an increase in cell metabolic activity. To probe these further, a set of experiments were conducted using a larger channel with a height of 50 µm for the larger cell types (MSCs and MG63s), while maintaining the same flow rate of 10 µL min^−1^. This effectively reduces the wall effects on the cell but also reduces the average velocity of the cells meaning that the duration of acoustic exposure is doubled. Based on this, we expected to see an increase of the acoustic‐based effects, particularly if hypothesis 2 of a direct influence of acoustic excitation upon metabolic rate is correct (the migration of hypothesis 1 can be expected to occur quickly so exposure time would not play a role).

The smaller L929 and HaCaT cells were again passed through the initial 25 µm height channel, but with the flow rate halved to 5 µL min^−1^ to lower the shear stresses and increase the exposure time. If hypothesis one regarding a shear‐induced effect is correct, we should see little change, as the acoustics act to reverse the shear effects. However the hypothesis of a direct effect of acoustic stimulation on cellular metabolism is correct, we would expect to see a further increase in the metabolic rate for cells exposed to ultrasound for longer. The associated velocities and shear stresses within these channels are numerically solved and reported in the Supplementary Information (see Figure S2, Supporting Information).

### 3.5 Untangling the Effects of Shear from Acoustic Stimulation on MSCs and MG63

**Figure 6:**
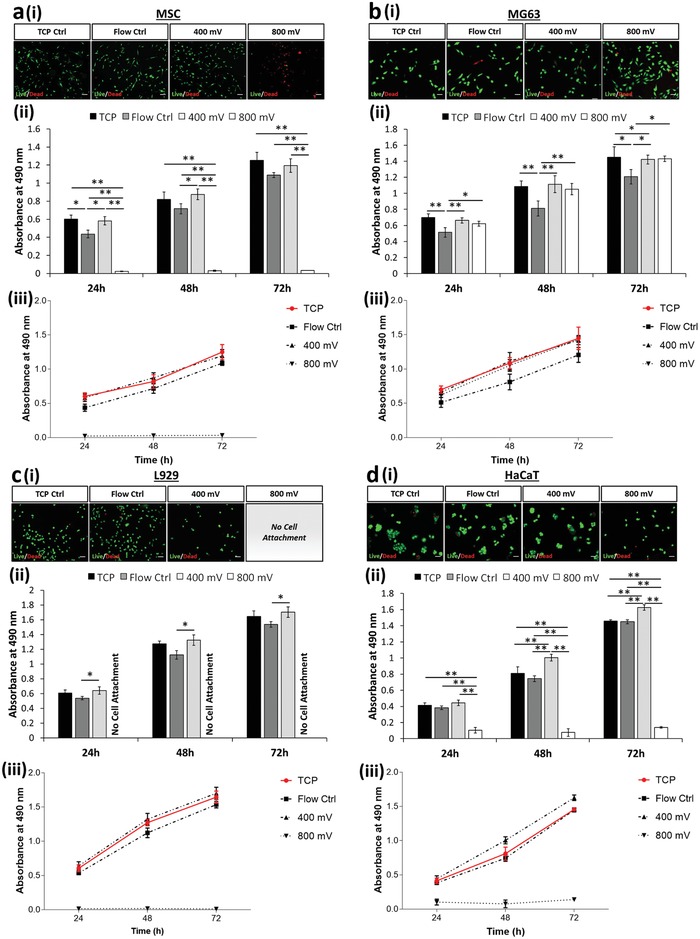
Increasing channel dimensions (i.e., 50 µm channel height) presents enhanced acoustic effects while enabling a) MSC and b) MG63 cell extraction. Decreasing the flow rate (i.e., 5 µL min^−1^) modulates metabolic activity of c) L929 and d) HaCaT while retaining a high degree of cell viability. a–d) Live/dead (green/red) fluorescence staining i) 24 h postexposure (see Tables S2 and S3 in the Supporting Information for tabulated live cell percentage) and ii) formazan absorbance (MTS assay; metabolic activity) are presented. MTS assay data analyzed using one‐way ANOVA with Tukey post hoc testing. iii) Growth rate extracted from MTS data is presented for all cell lines. All data presented as mean ± SD from triplicate samples (*n* = 9). Scale bar, 50 µm. Statistically different samples are denoted by *p < 0.05, **p < 0.005.

Using the modified conditions, experiments using the MTS assay and live/dead staining were conducted, again comparing cells plated directly onto TCP to cells exposed to flow‐only and those under acoustic stimulation. An increase of the channel height to 50 µm, effectively reducing wall effects on the cells, resulted in a full set of data, mitigating issues related to cell attachment as observed in Figure 4. A consistently high percentage of viable cells was observed using a live/dead staining, for both MSCs and MG63s, with the exception of MSCs stimulated with 800 mV in which a large proportion (93 ± 6%) of cell death was observed (**Figure** **6**a‐i and Table S2, Supporting Information). This can be attributed to excessive acoustic excitation, in terms of the input power and exposure time, effectively identifying the upper limit of exposure. MSCs are widely known to be extremely mechanosensitive,^67^, ^68^ thus unsurprisingly show the lowest tolerance to a persistent external stress from the cell lines investigated here. As expected, due to the low proportion of live cells, the metabolic activity for the 800 mV acoustic exposure, across all three time points is significantly reduced. It is important to note that although exposure below 800 mV does not result in cell death, cell health is not necessarily unaffected. Strikingly, and consistent with the initial experiments (Figure 4a‐ii,b‐ii), a trend of decreased metabolic activity was observed for the flow control condition and an increased metabolic activity in cells exposed to acoustic excitation was reported (Figure 6a‐ii,b‐ii). All stimulated cell populations had higher metabolic activity than those exposed to flow alone (Figure 6 a‐iii,b‐iii), with the exception of the 800 mV—treated MSCs in which the majority of the cell population was dead.

### 3.6 Untangling the Effects of Shear from Acoustic Stimulation on L929 and HaCaT

To understand the effects of reduced shear and extended acoustic exposure, the L929 and HaCaT cell lines were passed through the system at a reduced flow rate of 5 µL min^−1^. The expected exposure time was thus increased to 19 s, similar to that of the modified MSC and MG63 channels reported in Figure 6a,b. Consequentially, the flow control experimental results indicate a consistently low cell mortality rate (Figure 6c‐i,d‐i) and depict a reduced variation in metabolic activity, relative to the TCP control (Figure 6c‐i–iii,d‐i–iii), when compared to the data presented in Figure 4c‐ii,d‐ii, suggesting that the effects of shear induced by the flow are relatively insignificant and that any variations observed are likely caused by the acoustic excitation.

L929s were not able to adhere after exposure to 800 mV stimulation—this suggests that the limit of tolerance to acoustic exposure was reached under these conditions. While a living HaCaT population was retrieved, as shown by live/dead staining (Figure 6d‐i and Table S3, Supporting Information), the significant drop in metabolic activity (Figure 6d‐ii–iii) can be attributed to an excessive stress inflicted by the prolonged exposure of higher acoustic intensities, rendering the cessation of normal biological function while preserving the cell membrane and structure. This ability to preserve its structural integrity can be attributed to the high levels of keratin in HaCaT cells^65^, ^66^, ^69^ that act to safeguard the cell under their normal function in the skin which can be exposed to harsh environments. Acting as double‐edged sword in this instance, the higher density of keratin renders the cell relatively stiff. We propose that this stiffness results in an increased exertion of acoustic radiation, due to the inherently higher acoustic contrast factor, making it more susceptible to an acoustic stimuli.^44^ This observation is potentially the clearest indication of the shortfall presented by simplistic live/dead assay as evidence for cell viability. Here, the reported live/dead percentage would serve as a “false positive” when used to substantiate cell viability in an acoustic microfluidic platform as it is contrasting in nature when compared to the cell metabolic activity.

The other notable pattern in this data are that both cell types again showed increased metabolic activity at 400 mV acoustic stimulation (Figure 6c‐iii,d‐iii). Especially the HaCaT cell type (Figure 6d‐ii–iii), which reported a higher metabolic activity than that of the TCP control. Overall, this leads to a pattern where flow appears to moderately decrease the metabolic activity but acoustic stimulation either mitigates this effect or overrides it by independently increasing the metabolic activity. The latter supports hypothesis 2, linking a mechanotransductive mechanism to ultrasonic excitation.

### 3.7 Flow Control and Acoustic Comparison

**Figure 7:**
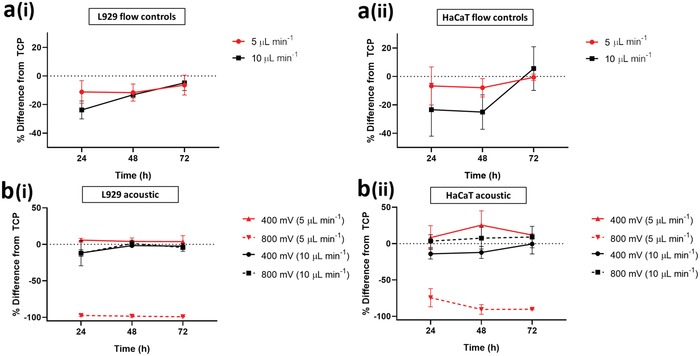
A percentage comparison between the high flow rate (i.e., 10 µL min^−1^; in black) and the low flow rate (i.e., 5 µL min^−1^; in red) settings normalized to the TCP control. a) Flow control comparisons for i) L929 and ii) HaCaT at different flow rates. b) Varying levels of acoustic exposure for i) L929 and ii) HaCaT at different flow rates.

As a further investigation of the effects of flow versus acoustic stimulation, the changes relative to the TCP control were calculated (**Figure** **7**). We observed a smaller reduction in the normalized metabolic activity (percentage difference to TCP control) at the lower flow rate for both cell L929 (Figure 7a‐i) and HaCaT cells (Figure 7a‐ii) at 24 h postexposure. Any differences in metabolic activity were diminished after 72 h (i.e., 0%; indicated by the horizontal dash line), indicating that the effects are caused by an acute stress that is recoverable. This reaffirms the hypothesis that shear stress experienced by the cell decreases the metabolic activity.

We next compared the impact of acoustic exposure, independently to flow rate. The 400 mV acoustic exposure settings consistently showed a higher metabolic rate than the TCP, for both flow rates (Figure 7b). When the detrimental effects of shear are lessened by operating at a lower flow rate and the length of exposure to acoustic effects is increased, the increase in metabolic rate is such that the values recorded are higher than those of the TCP. This offers firm support of hypothesis 2 whereby acoustic exposure has an impact on the cell metabolic activity.

## 4 Discussion

Here we fabricated a system that facilitates assessment of cell behavior following acoustic exposure and used a wide range of methodologies to examine the impact upon cell behavior. The results demonstrate sensitivity to acoustic exposure in a manner that is highly context‐dependent. Metabolic rate was seen to increase (benchmarked against a TCP control), cell adhesion prevented and morphology changed—all depending upon the conditions used and cell lines studied. Importantly, many of these occurred when no difference was seen in live/dead assays, highlighting the urgent need for a more nuanced approach when evaluating the biological impact of acoustic exposure.

**Figure 8:**
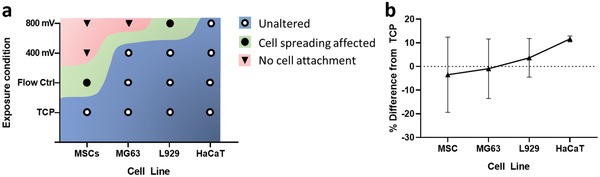
a) Description of distinct cell fate in terms of morphological changes as a function of cell line and exposure condition as the cells were passed through the 25 µm high channel device at 10 µL min^−1^ (see Figures 2 and 3 and Figure S3, Supporting Information). b) Percentage difference in cell metabolic activity normalized to TCP as a function of cell line with increasing stiffness based on results reported in Figure 6 at a 400 mV acoustic exposure at 72 h postexposure.

Based on the cell morphology data (Figures 2 and 3), we show adverse effects under some acoustic conditions in which cells were unable to attach or spread less effectively, so altering critical adherent cell functionality. This occurred to varying degrees, ranging from cells that could adhere to the substrate, but not spread (for the case of L929 cells; Figure 2) to cells that showed a complete inability to adhere to the substrate (e.g., MSCs as in Figure S3a and MG63 in Figure S3b, Supporting Information). As shown in **Figure** **8**a, we postulate a link with cell stiffness. As stiffness increases, we observe a better resistance to morphological changes resulting from the exposure conditions tested. The highly mechanosensitive cell considered, MSCs are more susceptible to external stressors, the MG63 cells slightly less so, both of which succumbed to cell attachment issues arising from acoustic exposures (see Figure 3, Figure S3a,b, Supporting Information) when passed through the 25 µm high channel at 10 µL min^−1^. This trend continues with the stiffer, L929 cells which succumbed to alteration in cell aspect ratio postexposure (i.e., cell spreading was affected) but successfully attached (Figure 3c). Finally, the stiffest cell considered, HaCaT was unaffected by all exposure conditions tested.

A similar trend is suggested for the reported variations in metabolic activity as a function of cell stiffness. We observed variations in the cell metabolic activity as a result of shear induced by flow as well as acoustic exposure, and showed clearly that this was not due to a change in absolute cell number from altered proliferation rates. First, the cellular metabolic activity was suppressed as a result of shear stress induced by the flow. Second and more importantly, we report an increase in cellular metabolic activity as a direct result of acoustic exposure (see Figure 6d‐ii–iii) for the first time. However, a further increase in acoustic exposure beyond a threshold (cell line‐dependent) results in a detrimental effect. This either 1) directly compromises the cells membrane, effectively killing the cells as in the case of MSCs (Figure 6a‐i), or 2) stresses the cells excessively such that the cell's metabolic activity is significantly suppressed, potentially due to the cessation of normal biological function (Figure 6d‐ii–iii), in the absence of changes to structural integrity. The latter occurs while measurements also yield a low mortality percentage, showing the inadequacy of using live/dead assays as a single readout. Stamp et al.^70^ observed enhanced cell migration within a tissue under the influence of ultrasonic excitation. In this context they hypothesized possible mechanisms including mechanical actuation, better delivery of nutrients, and thermal and electrical effects. While this study does not deal with metabolic activity of individual cells, and the conditions within our microfluidic channel are more easily controlled eliminating some of the possible mechanisms, others could potentially apply. Furthermore, we suggest a link with cell stiffness. A trend of increasing metabolic activity as the cell stiffness increases was observed, (Figure 8b). Here, the percentage difference in metabolic activity as compared to TCP is reported. This is consistent with an increase in the resultant acoustic radiation force as the stiffness of the suspended matter increases.^44^, ^64^ Alternatively, we hypothesize that the cells are actively attempting to improve resistance to an external stress by increasing their structural stiffness. The MTS assay used as a surrogate marker of metabolism functions through a formazan reduction accomplished by nicotinamide adenine dinucleotide phosphate (NADPH) or nicotinamide adenine dinucleotide (NADH).^71^ Due to NADPH's link to the regulation of cholesterol synthesis,^72^ we could assume that our findings may be a result of cell stiffness increase in response to acoustic exposure. Studies suggesting roles for cholesterol in cell membrane stiffness and tension have been conducted;^73^ however, further investigation would be required to validate these theories. Interestingly, the data variability of each cell consistently decreases as cell stiffness increases, showing the smallest variance in HaCaT (10 kPa^59^) and L929 (4 kPa^74^) and larger variance for the softer MG63 and MSCs (0.3–0.8 kPa^57^, ^58^) respectively (see Table S4 in the Supporting Information for tabulated values). This is indicative of the inherent heterogeneity which is known to be more prevalent in softer cell types (i.e., MSCs) than stiffer ones (i.e., HaCaT).

## 5 Conclusion

In conclusion, our data clearly show differences in cell behavior in response to acoustic stimulation, aspects that are not evident when using the standard live/dead stain as a single readout of biocompatibility. This has critical implications for the methodologies used to evaluate the biocompatibility of acoustofluidic devices and platforms and makes a strong case for the inclusion of a broad panel of cellular readouts. The variations and chronic thresholds in response are cell type‐specific and so safe operation ranges should be considered while developing acoustic based microfluidic platforms with reference to the cell type used. Our findings also reveal a tantalizing hint toward a correlation between acoustic exposure, cell stiffness and cellular metabolism, which if understood, could be harnessed for therapeutic applications in the future.

## 6 Experimental Section

*Cell Culture*: Mesenchymal Stromal Cells (human, bone marrow derived, Lonza) and MG63 (human osteosarcoma, ATCC) cells were maintained in Dulbecco's modified Eagle medium (DMEM) containing [1 g L^−1^] d‐glucose and [110 mg L^−1^] sodium pyruvate (Life Technologies), supplemented with [100 U mL^−1^] penicillin‐streptomycin (Life technologies) and 10% (v/v) fetal bovine serum (FBS) (Scientifix). HaCaT (human keratinocyte, ATCC) and L929 (mouse fibroblast, ATCC) cells were maintained in DMEM containing [4.5 g L^−1^] d‐glucose and [110 mg L^−1^] sodium pyruvate (Life Technologies), supplemented with [100 U mL^−1^] penicillin‐streptomycin (Life technologies) and 10% (v/v) FBS (Scientifix). Maintenance was undertaken in humidified conditions at 37 °C with 5% CO_2_. All cells were routinely screened for and confirmed free of mycoplasma every 3 months. 24 h prior to experimentation, cells were serum‐starved overnight in standard culture media supplemented with only 0.25% FBS.

*Acoustic Exposure*: The IDTs were designed such that the SAW wavelength, λ_SAW_ at the desired frequency was twice the pitch of the electrodes. Here a wavelength of 80 µm was used, resulting in an operational frequency of 48.5 MHz. The λ_SAW_ was selected such that the half acoustic wavelength, λ_ac_, in fluid was approximately the size of the cells considered (i.e., λ_ac_/2 = 15 µm). Energy coupled into the fluid was lost from the substrate wave; hence, a pair of IDTs was used to offer a more uniform field than could be expected from a single IDT. Following serum starvation, cells were lifted using TrypLE Express (LifeTech), collected at the desired density and transferred to microfluidic devices for exposure. Cells were maintained at a constant flow rate of 5 or 10 µL min^−1^ as per the experimental data set, while being excited at the designed frequency and power of either 400 mV (500 mW; accommodating for amplification via power amplifier and s11 values) or 800 mV (1375 mW; accommodating for amplification via power amplifier and corresponding s11 values) typical of similar SAW‐based manipulation platforms at these frequencies, accommodating for the flow rates.^6^, ^28^, ^60^, ^75^, ^76^ To circumvent issues related to clogging, cell aggregation was avoided via frequent agitation of the syringe and a 180° rotation every 3 min to avoid sedimentation. To minimize any external influences, the chip was actively cooled to maintain its temperature via the aid of a peltier cooler, accompanied by a heat sink and fan. The cell numbers across each experimental run were maintained at either 2500 cells cm^−2^ (MSCs and MG63 cells) or 8000 cells cm^−2^ (HaCaT and L929 cells) throughout.

*MTS Metabolic Assay*: Metabolic activity was screened using a CellTiter 96 AQueous One Solution assay kit (Promega, USA) with a staining solution made up according to the manufacturer instructions. MTS assays were conducted 24, 48, and 72 h following exposure in which cells were rinsed with phosphate‐buffered saline (PBS) and left in MTS staining solution for 3 h. Solutions were then removed and absorbance quantified at 490 nm using a Multiskan spectrum plate reader (Thermo).

*Live/Dead Staining Assay*: Cell viability was assessed using a live/dead assay kit (Life Technologies, USA) with a staining solution made up according to the manufacturer instructions. Live/dead staining was conducted at time points of 24 h postexposure. Samples were imaged using a Nikon eclipse Ts2 microscope followed by further quantitative analysis using ImageJ. Viability was calculated using the following formula: (live cell count / (live cell count + dead cell count)) × 100.

*Immunofluorescence Staining*: To validate cell proliferation alongside cell/nuclear morphology changes, immunofluorescence staining was performed. Cell monolayers were washed with PBS and fixed in 4% Paraformaldehyde (PFA) (Sigma, USA) diluted in PBS for 30 min at room temperature (RT). Cells were permeabilized with 0.5% Triton X‐100 (Sigma, USA) diluted in PBS for 15 min, then blocked in 3% (w/v) bovine serum albumen (BSA) diluted in PBS for 30 min. Cells were then incubated in primary antibody (Ki67, AbCam [1:1000]), diluted in blocking solution for 1 h at RT. Cells were subsequently washed 3 × 5 min in PBS and further incubated in a mixture of secondary antibody (antimouse alexa fluor 555, Sigma, USA), Actin‐green (Life Technologies, USA) and Hoechst 33 342 (Life Technologies, USA) at a dilution of 1:300, 2 drops mL^−1^ and 1:2000 respectively for 1 h at RT. Images were taken as described for live/dead staining assays.

*Quantification of Cell and Nuclear Phenotypes*: For the assessment of both cell and nuclear phenotypes, immunofluorescence images counterstained with Phalloidin (cell phenotypes) and Hoechst (nuclear phenotypes) were processed in ImageJ. Images were thresholded, counted, and analyzed using the shape descriptors plugin, with analysis parameters maintained as to enable direct comparisons between conditions. Cell area and aspect ratios provide detailed information on cell shape and orientation in 2D, whereas nuclear circularity index is a measure of how closely the nuclear shape resembles that of a mathematically perfect circle (scale from 0 to 1, with the perfect circle denoted as 1). Nuclear circularity index of < 0.5 are potential indicators of a diseased state, e.g., nuclear blebbing in cancer or progeria development.

*Statistical Analysis*: A Kolmogorov–Smirnov test was used to test data for normal distribution and Levene's test was used to determine homogeneity of variance. Data with a normal distribution were analyzed by one‐way ANOVA and Tukey (equal variance) or Games‐Howell (unequal variance) post hoc tests. Nonparametric data were analyzed by Kruskal–Wallis test unless otherwise stated. All statistical analysis was performed using GraphPad Prism v7.

## Conflict of Interest

The authors declare no conflict of interest.

## Supporting information

Supporting Information

## References

[1] A. R. Wheeler , W. R. Throndset , R. J. Whelan , A. M. Leach , R. N. Zare , Y. H. Liao , K. Farrell , I. D. Manger , A. Daridon , Anal. Chem. 2003, 75, 3581. 14570213 10.1021/ac0340758

[2] K. J. Regehr , M. Domenech , J. T. Koepsel , K. C. Carver , S. J. Ellison‐Zelski , W. L. Murphy , L. A. Schuler , E. T. Alarid , D. J. Beebe , Lab Chip 2009, 9, 2132. 19606288 10.1039/b903043c PMC2792742

[3] S. M. Prakadan , A. K. Shalek , D. A. Weitz , Nat. Rev. Genet. 2017, 18, 345. 28392571 10.1038/nrg.2017.15 PMC5495114

[4] W. Lee , P. Tseng , D. Di Carlo , in Microtechnology for Cell Manipulation and Sorting (Eds: Lee W., Tseng P., Di Carlo) D., Springer International Publishing, Cham 2017, pp. 1–14.

[5] D. Di Carlo , Lab Chip 2009, 9, 3038. 19823716 10.1039/b912547g

[6] D. J. Collins , B. Morahan , J. Garcia‐Bustos , C. Doerig , M. Plebanski , A. Neild , Nat. Commun. 2015, 6, 8686. 26522429 10.1038/ncomms9686 PMC4659840

[7] J. V. Sweedler , E. A. Arriaga , Anal. Bioanal. Chem. 2006, 387, 1.

[8] N. W. Choi , M. Cabodi , B. Held , J. P. Gleghorn , L. J. Bonassar , A. D. Stroock , Nat. Mater. 2007, 6, 908. 17906630 10.1038/nmat2022

[9] H. Andersson , A. van den Berg , Lab Chip 2004, 4, 98. 15052347 10.1039/b314469k

[10] Y. Chen , L. Huang , K. Ren , X. Shi , H. Wu , in RSC Smart Materials; The Royal Society of Chemistry (Ed: Wang Q.), RSC Publishing, Cambridge, UK 2017, pp. 596–614.

[11] F. S. Frueh , M. D. Menger , N. Lindenblatt , P. Giovanoli , M. W. Laschke , Crit. Rev. Biotechnol. 2017, 37, 613. 27439727 10.1080/07388551.2016.1209157

[12] A. Khademhosseini , R. Langer , J. Borenstein , J. P. Vacanti , Proc. Natl. Acad. Sci. USA 2006, 103, 2480. 16477028 10.1073/pnas.0507681102 PMC1413775

[13] S. Faley , K. Seale , J. Hughey , D. K. Schaffer , S. VanCompernolle , B. McKinney , F. Baudenbacher , D. Unutmaz , J. P. Wikswo , Lab Chip 2008, 8, 1700. 18813394 10.1039/b719799c PMC4160168

[14] I. K. Zervantonakis , C. R. Kothapalli , S. Chung , R. Sudo , R. D. Kamm , Biomicrofluidics 2011, 5, 013406. 10.1063/1.3553237 PMC3082343 21522496

[15] X. Ding , S.‐C. S. Lin , M. I. Lapsley , S. Li , X. Guo , C. Y. Chan , I.‐K. Chiang , L. Wang , J. P. McCoy , T. J. Huang , Lab Chip 2012, 12, 4228. 22992833 10.1039/c2lc40751e PMC3956451

[16] P. R. C. Gascoyne , J. Vykoukal , Electrophoresis 2002, 23, 1973. 12210248 10.1002/1522-2683(200207)23:13<1973::AID-ELPS1973>3.0.CO;2-1 PMC2726256

[17] H. Ahmed , G. Destgeer , J. Park , J. H. Jung , H. J. Sung , Adv. Sci. 2018, 5, 1700285. 10.1002/advs.201700285 PMC5827645 29619294

[18] E. Brouzes , M. Medkova , N. Savenelli , D. Marran , M. Twardowski , J. B. Hutchison , J. M. Rothberg , D. R. Link , N. Perrimon , M. L. Samuels , Proc. Natl. Acad. Sci. USA 2009, 106, 14195. 19617544 10.1073/pnas.0903542106 PMC2732882

[19] D. W. Inglis , J. A. Davis , R. H. Austin , J. C. Sturm , Lab Chip 2006, 6, 655. 16652181 10.1039/b515371a

[20] J. A. Davis , D. W. Inglis , K. J. Morton , D. A. Lawrence , L. R. Huang , S. Y. Chou , J. C. Sturm , R. H. Austin , Proc. Natl. Acad. Sci. USA 2006, 103, 14779. 17001005 10.1073/pnas.0605967103 PMC1595428

[21] J.‐C. Baret , O. J. Miller , V. Taly , M. Ryckelynck , A. El‐Harrak , L. Frenz , C. Rick , M. L. Samuels , J. B. Hutchison , J. J. Agresti , D. R. Link , D. A. Weitz , A. D. Griffiths , Lab Chip 2009, 9, 1850. 19532959 10.1039/b902504a

[22] D. W. Inglis , R. Riehn , R. H. Austin , J. C. Sturm , Appl. Phys. Lett. 2004, 85, 5093.

[23] N. Xia , T. P. Hunt , B. T. Mayers , E. Alsberg , G. M. Whitesides , R. M. Westervelt , D. E. Ingber , Biomed. Microdevices 2006, 8, 299. 17003962 10.1007/s10544-006-0033-0

[24] Y. Wang , Y. Zhao , S. K. Cho , J. Micromech. Microeng. 2007, 17, 2148.

[25] M. P. MacDonald , G. C. Spalding , K. Dholakia , Nature 2003, 426, 421. 14647376 10.1038/nature02144

[26] B. Landenberger , H. Höfemann , S. Wadle , A. Rohrbach , Lab Chip 2012, 12, 3177. 22767208 10.1039/c2lc21099a

[27] H. Shafiee , M. B. Sano , E. A. Henslee , J. L. Caldwell , R. V. Davalos , Lab Chip 2010, 10, 438. 20126683 10.1039/b920590j

[28] D. J. Collins , C. Devendran , Z. Ma , J. W. Ng , A. Neild , Y. Ai , Sci. Adv. 2016, 2, e1600089. 27453940 10.1126/sciadv.1600089 PMC4956186

[29] A. Ozcelik , J. Rufo , F. Guo , Y. Gu , P. Li , J. Lata , T. J. Huang , Nat. Methods 2018, 15, 1021. 30478321 10.1038/s41592-018-0222-9 PMC6314293

[30] A. Marzo , S. A. Seah , B. W. Drinkwater , D. R. Sahoo , B. Long , S. Subramanian , Nat. Commun. 2015, 6, 8661. 26505138 10.1038/ncomms9661 PMC4627579

[31] C. Devendran , T. Albrecht , J. Brenker , T. Alan , A. Neild , Lab Chip 2016, 16, 3756. 27722363 10.1039/c6lc00798h

[32] A. Fakhfouri , C. Devendran , A. Ahmed , J. Soria , A. Neild , Lab Chip 2018, 18, 3926. 30474095 10.1039/c8lc01155a

[33] A. Fakhfouri , C. Devendran , D. J. Collins , Y. Ai , A. Neild , Lab Chip 2016, 16, 3515. 27458086 10.1039/c6lc00590j

[34] G. Destgeer , B. H. Ha , J. Park , J. H. Jung , A. Alazzam , H. J. Sung , Anal. Chem. 2015, 87, 4627. 25800052 10.1021/acs.analchem.5b00525

[35] C. Devendran , N. R. Gunasekara , D. J. Collins , A. Neild , RSC Adv. 2016, 6, 5856.

[36] X. Ding , Z. Peng , S.‐C. S. Lin , M. Geri , S. Li , P. Li , Y. Chen , M. Dao , S. Suresh , T. J. Huang , Proc. Natl. Acad. Sci. USA 2014, 111, 12992. 25157150 10.1073/pnas.1413325111 PMC4246961

[37] K. Melde , A. G. Mark , T. Qiu , P. Fischer , Nature 2016, 537, 518. 27652563 10.1038/nature19755

[38] D. Ahmed , A. Ozcelik , N. Bojanala , N. Nama , A. Upadhyay , Y. Chen , W. Hanna‐Rose , T. J. Huang , Nat. Commun. 2016, 7, 11085. 27004764 10.1038/ncomms11085 PMC4814581

[39] G. Destgeer , K. H. Lee , J. H. Jung , A. Alazzam , H. J. Sung , Lab Chip 2013, 13, 4210. 23982077 10.1039/c3lc50451d

[40] L. Meng , X. Liu , Y. Wang , W. Zhang , W. Zhou , F. Cai , F. Li , J. Wu , L. Xu , L. Niu , H. Zheng , Adv. Sci. 2019, 6, 1900557. 10.1002/advs.201900557 PMC6724477 31508275

[41] P. Huang , S. Zhao , H. Bachman , N. Nama , Z. Li , C. Chen , S. Yang , M. Wu , S. P. Zhang , T. J. Huang , Adv. Sci. 2019, 6, 1900913. 10.1002/advs.201900913 PMC6774021 31592417

[42] H. Van Phan , M. B. Coskun , M. Sesen , G. Pandraud , A. Neild , T. Alan , Lab Chip 2015, 15, 4206. 26381355 10.1039/c5lc00836k

[43] Y. Zhang , C. Devendran , C. Lupton , A. de Marco , A. Neild , Lab Chip 2019, 19, 262. 30564824 10.1039/c8lc01117f

[44] P. Augustsson , J. T. Karlsen , H.‐W. Su , H. Bruus , J. Voldman , Nat. Commun. 2016, 7, 11556. 27180912 10.1038/ncomms11556 PMC4873643

[45] O. Manneberg , B. Vanherberghen , J. Svennebring , H. M. Hertz , B. Önfelt , M. Wiklund , Appl. Phys. Lett. 2008, 93, 063901.

[46] J. Hultström , O. Manneberg , K. Dopf , H. M. Hertz , H. Brismar , M. Wiklund , Ultrasound Med. Biol. 2007, 33, 145. 17189057 10.1016/j.ultrasmedbio.2006.07.024

[47] S. Li , P. Glynne‐Jones , O. G. Andriotis , K. Y. Ching , U. S. Jonnalagadda , R. O. C. Oreffo , M. Hill , R. S. Tare , Lab Chip 2014, 14, 4475. 25272195 10.1039/c4lc00956h PMC4227593

[48] M. A. Burguillos , C. Magnusson , M. Nordin , A. Lenshof , P. Augustsson , M. J. Hansson , E. Elmér , H. Lilja , P. Brundin , T. Laurell , T. Deierborg , PLoS One 2013, 8, e64233. 23724038 10.1371/journal.pone.0064233 PMC3664584

[49] W. L. Ung , K. Mutafopulos , P. Spink , R. W. Rambach , T. Franke , D. A. Weitz , Lab Chip 2017, 17, 4059. 28994439 10.1039/c7lc00715a

[50] T. Franke , S. Braunmuller , L. Schmid , A. Wixforth , D. A. Weitz , Lab Chip 2010, 10, 789. 20221569 10.1039/b915522h

[51] S. M. Naseer , A. Manbachi , M. Samandari , P. Walch , Y. Gao , Y. S. Zhang , F. Davoudi , W. Wang , K. Abrinia , J. M. Cooper , A. Khademhosseini , S. R. Shin , Biofabrication 2017, 9, 015020. 28195834 10.1088/1758-5090/aa585e PMC5421404

[52] P. W. S. Pui , F. Trampler , S. A. Sonderhoff , M. Groeschl , D. G. Kilburn , J. M. Piret , Biotechnol. Prog. 1995, 11, 146. 7766098 10.1021/bp00032a005

[53] O. Doblhoff‐Dier , T. Gaida , H. Katinger , W. Burger , M. Groschl , E. Benes , Biotechnol. Prog. 1994, 10, 428. 7765096 10.1021/bp00028a600

[54] S. Varma , J. Voldman , Lab Chip 2018, 18, 3333. 30324208 10.1039/c8lc00746b PMC6254237

[55] L. Chau , M. Doran , J. Cooper‐White , Lab Chip 2009, 9, 1897. 19532965 10.1039/b823180j

[56] S. Regmi , A. Fu , K. Q. Luo , Sci. Rep. 2017, 7, 39975. 28054593 10.1038/srep39975 PMC5215453

[57] X. Wang , Y. Yang , X. Hu , N. Kawazoe , Y. Yang , G. Chen , Anal. Sci. 2016, 32, 1177. 27829622 10.2116/analsci.32.1177

[58] E. J. Mah , G. E. McGahey , A. F. Yee , M. A. Digman , Collagen Stiffness Modulates MDA‐MB231 Cell Metabolism Through Adhesion‐Mediated Contractility, 2018, 10.2139/ssrn.3188427. PMC6244401 30459440

[59] T. Kobiela , M. Milner‐Krawczyk , M. Pasikowska‐Piwko , K. Bobecka‐Wesołowska , I. Eris , W. Święszkowski , I. Dulinska‐Molak , Int. J. Pept. Res. Ther. 2018, 24, 577. 30416406 10.1007/s10989-017-9648-7 PMC6208634

[60] J. W. Ng , D. J. Collins , C. Devendran , Y. Ai , A. Neild , Microfluid. Nanofluid. 2016, 20.

[61] D. J. Collins , R. O'Rorke , C. Devendran , Z. Ma , J. Han , A. Neild , Y. Ai , Phys. Rev. Lett. 2018, 120, 074502. 29542954 10.1103/PhysRevLett.120.074502

[62] X. Ding , S.‐C. S. Lin , B. Kiraly , H. Yue , S. Li , I.‐K. Chiang , J. Shi , S. J. Benkovic , T. J. Huang , Proc. Natl. Acad. Sci. USA 2012, 109, 11105. 22733731 10.1073/pnas.1209288109 PMC3396524

[63] C. Devendran , D. J. Collins , Y. Ai , A. Neild , Phys. Rev. Lett. 2017, 118, 154501. 28452526 10.1103/PhysRevLett.118.154501

[64] P. B. Muller , R. Barnkob , M. J. H. Jensen , H. Bruus , Lab Chip 2012, 12, 4617. 23010952 10.1039/c2lc40612h

[65] M. Moravcová , A. Libra , J. Dvořáková , A. Víšková , T. Muthný , V. Velebný , L. Kubala , Interdiscip. Toxicol. 2013, 6, 203. 24678259 10.2478/intox-2013-0030 PMC3945759

[66] F. N. Kenny , Z. Drymoussi , R. Delaine‐Smith , A. P. Kao , A. C. Laly , M. M. Knight , M. P. Philpott , J. T. Connelly , J. Cell Sci. 2018, 131, jcs215780. 29669739 10.1242/jcs.215780

[67] J. E. Frith , G. D. Kusuma , J. Carthew , F. Li , N. Cloonan , G. A. Gomez , J. J. Cooper‐White , Nat. Commun. 2018, 9, 257. 29343687 10.1038/s41467-017-02486-0 PMC5772625

[68] A. J. Engler , S. Sen , H. L. Sweeney , D. E. Discher , Cell 2006, 126, 677. 16923388 10.1016/j.cell.2006.06.044

[69] D. J. Fu , C. Thomson , D. P. Lunny , P. J. Dopping‐Hepenstal , J. A. McGrath , F. J. D. Smith , W. H. Irwin McLean , D. M. Leslie Pedrioli , J. Invest. Dermatol. 2014, 134, 754. 23962810 10.1038/jid.2013.356 PMC3923277

[70] M. E. M. Stamp , M. S. Brugger , A. Wixforth , C. Westerhausen , Biomater. Sci. 2016, 4, 1092. 27138623 10.1039/c6bm00125d

[71] M. V. Berridge , A. S. Tan , Arch. Biochem. Biophys. 1993, 303, 474. 8390225 10.1006/abbi.1993.1311

[72] F. J. Byfield , H. Aranda‐Espinoza , V. G. Romanenko , G. H. Rothblat , I. Levitan , Biophys. J. 2004, 87, 3336. 15347591 10.1529/biophysj.104.040634 PMC1304801

[73] A. Biswas , P. Kashyap , S. Datta , T. Sengupta , B. Sinha , Biophys. J. 2019, 116, 1456. 30979551 10.1016/j.bpj.2019.03.016 PMC6486507

[74] H. W. Wu , T. Kuhn , V. T. Moy , Scanning 1998, 20, 389. 9737018 10.1002/sca.1998.4950200504

[75] D. J. Collins , T. Alan , A. Neild , Lab Chip 2014, 14, 1595. 24638896 10.1039/c3lc51367j

[76] J. W. Ng , C. Devendran , A. Neild , Lab Chip 2017, 17, 3489. 28929163 10.1039/c7lc00862g

