# Supplementary material for: Cell Adhesion, Morphology, and Metabolism Variation via Acoustic Exposure within Microfluidic Cell Handling Systems
Source: Adv Sci (Weinh). 2019 Oct 30;6(24):1902326. doi: 10.1002/advs.201902326 (PMC6918100; doi:10.1002/advs.201902326)
Supplement: Supplementary file 1 — Supporting Information [file ADVS-6-1902326-s001.pdf]

Copyright WILEY-VCH Verlag GmbH & Co. KGaA, 69469 Weinheim, Germany, 2019.

ADVANCED SCIENCE Open Access

Supporting Information

for *Adv. Sci.,* DOI: 10.1002/advs.201902326

Cell Adhesion, Morphology, and Metabolism Variation via Acoustic Exposure within Microfluidic Cell Handling Systems

*Citsabehsan Devendran, James Carthew, Jessica E. Frith, and Adrian Neild**

Supporting Information

Cell Adhesion, Morphology and Metabolism Variation via Acoustic Exposure within Microfluidic Cell Handling Systems

*Citsabehsan Devendran, James Carthew, Jessica E. Frith and Adrian Neild**

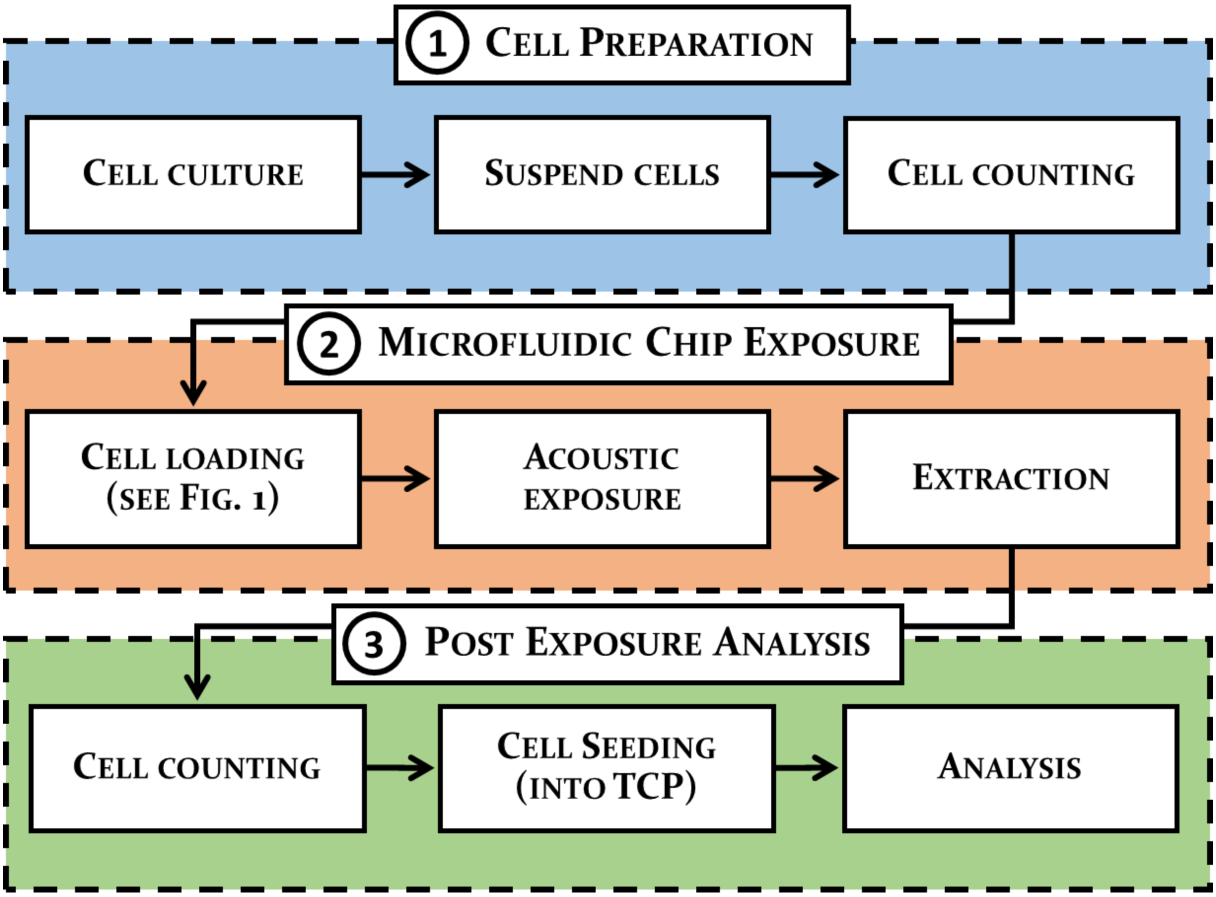

Figure S 1. The process flow diagram depicting the preparation of cells, exposure to varying acoustic conditions and subsequent analysis methods.

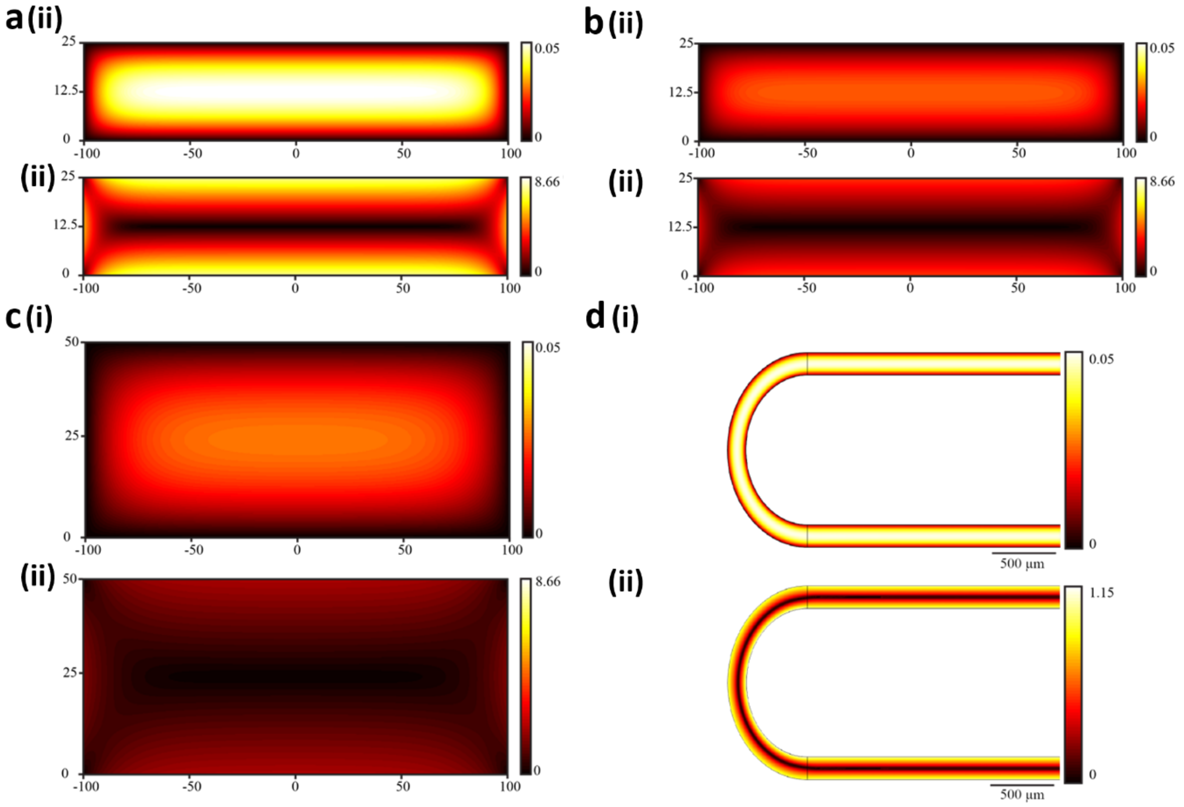

Figure S 2. Flow velocity (i) and shear profiles (ii) in the cross section view of the channel 25 µm deep channel at 10 µL min^{-1}(a), 25 µm deep channel at 5 µL min^{-1} (b) and 50 µm deep channel at 10 µL min^{-1} (c). The top view (d) of the flow velocity (i) and shear (ii) profile at the channel bend for a 25 µm deep channel at 10 µL min^{-1} scenario.

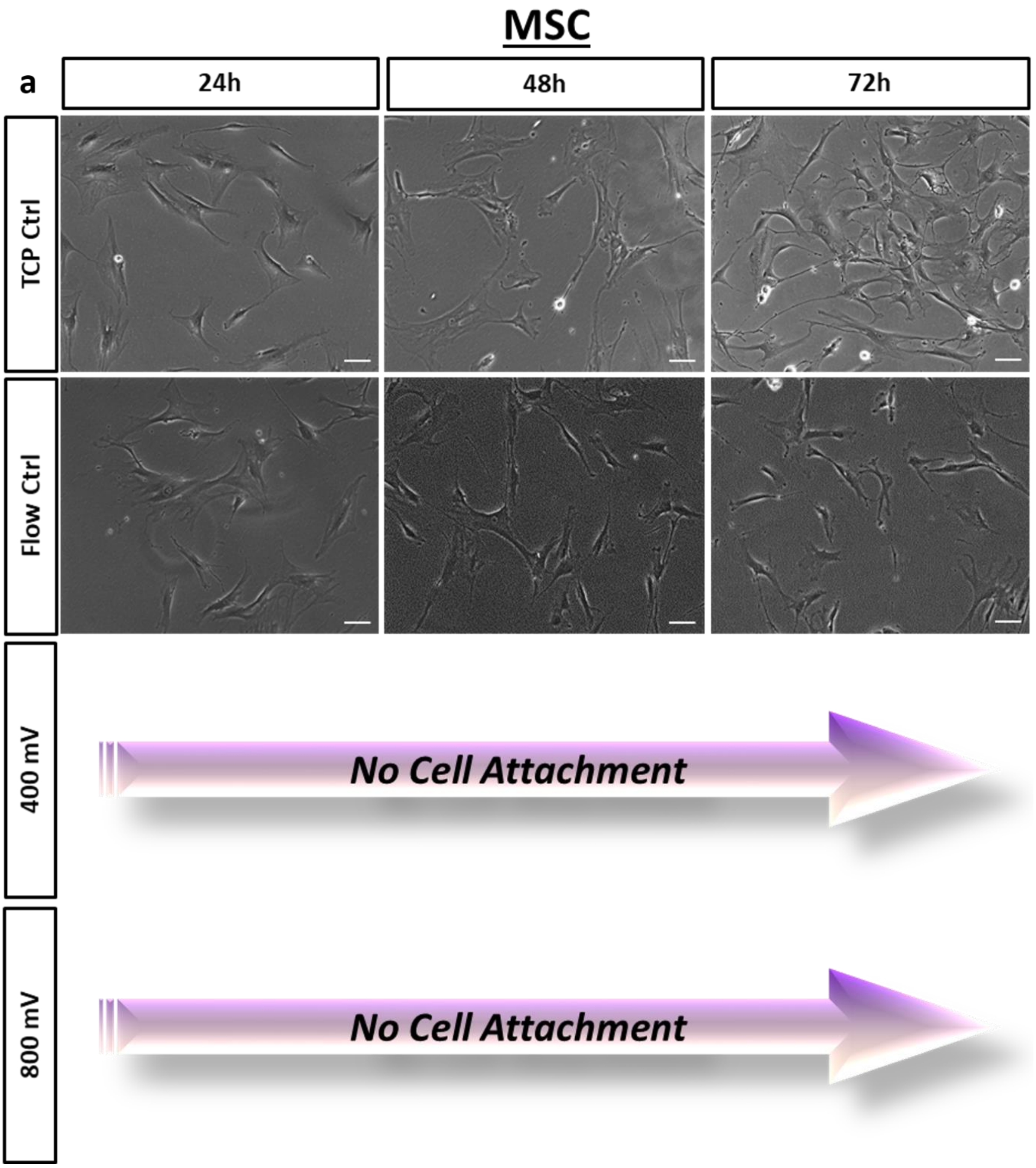
MSC
a
24h
48h
72h
TCP Ctrl
Flow Ctrl
400 mV
No Cell Attachment
800 mV
No Cell Attachment

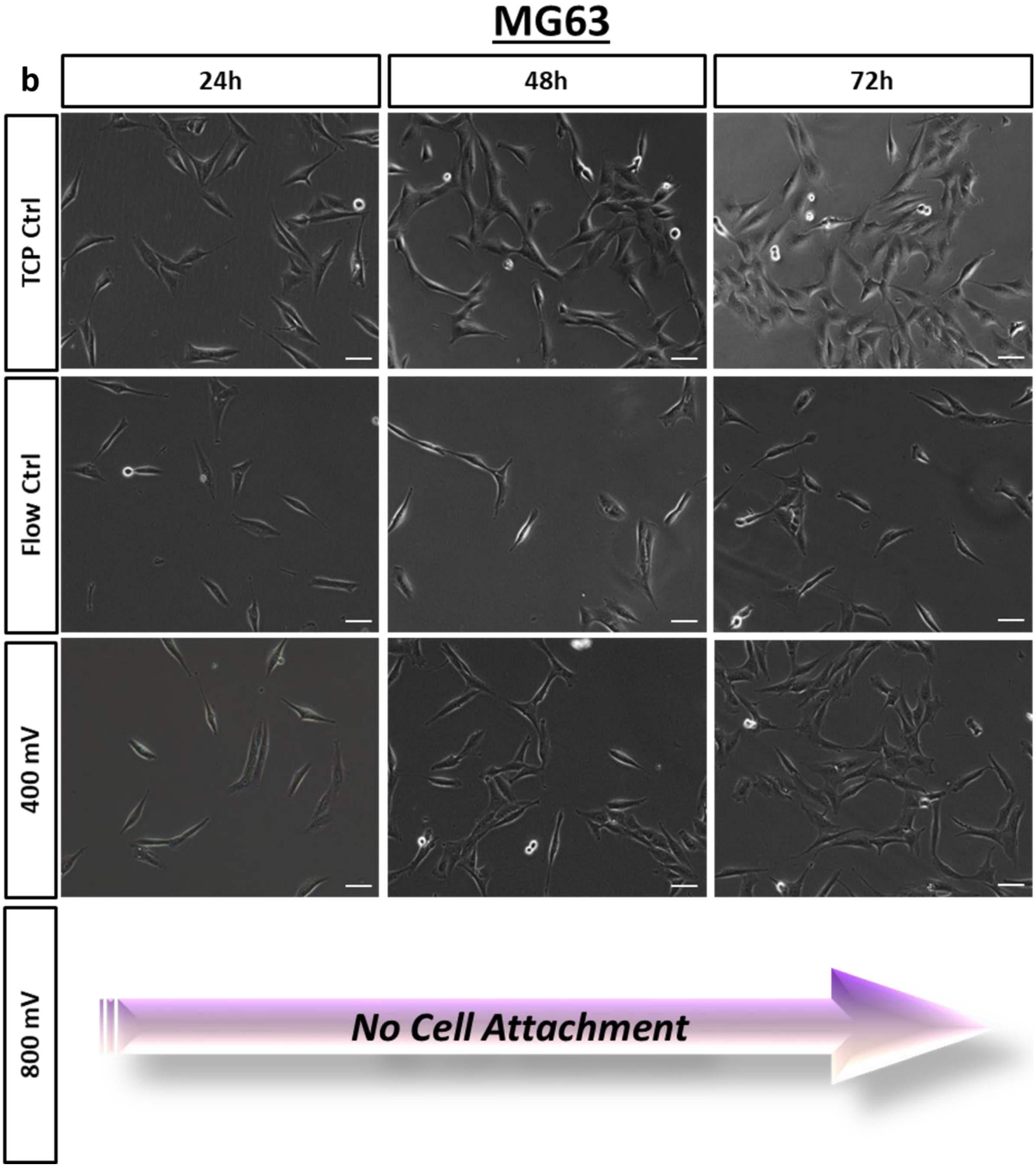
MG63
b
24h
48h
72h
TCP Ctrl
Flow Ctrl
400 mV
800 mV
No Cell Attachment

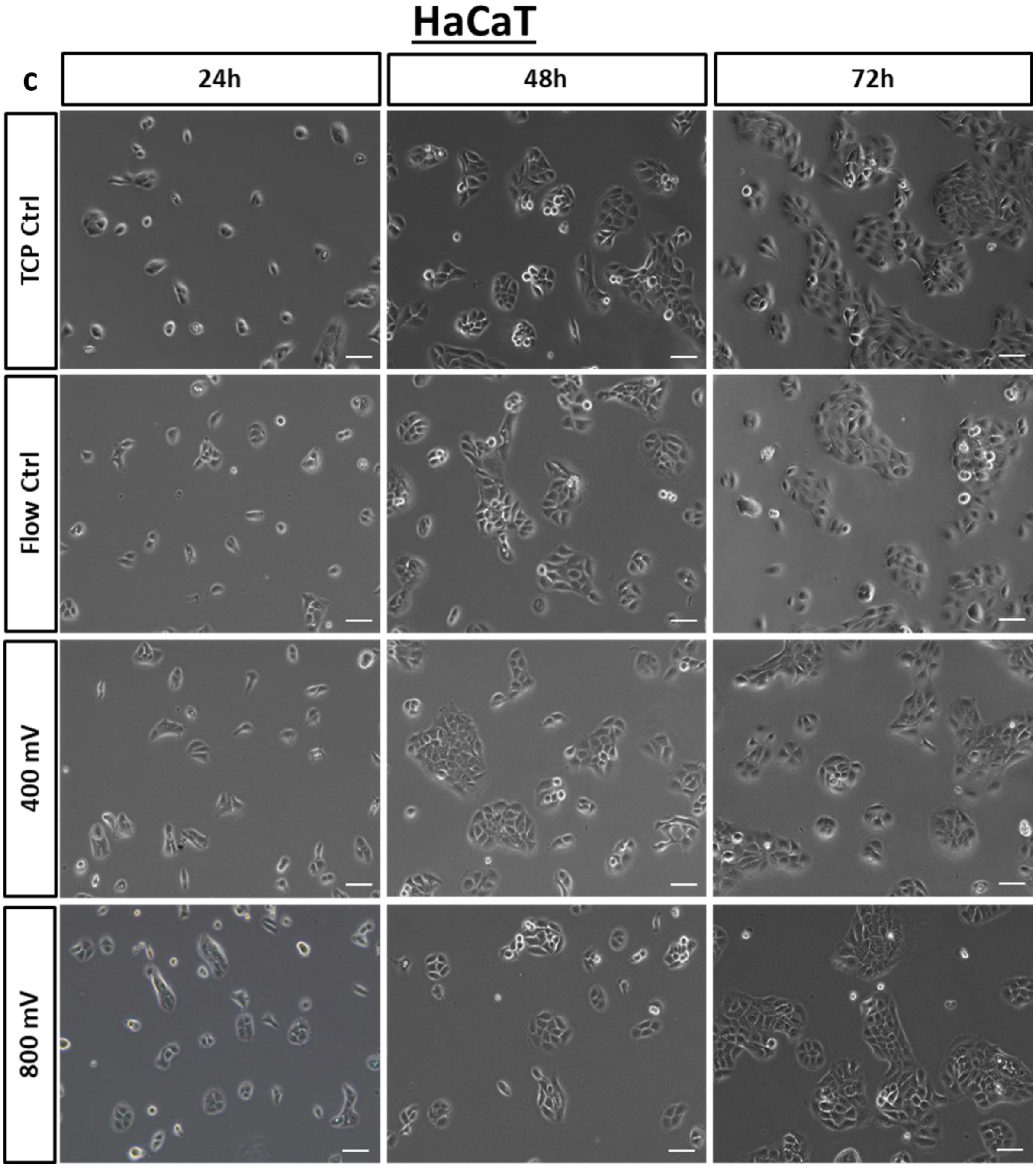

Figure S 3. Acoustic exposure resulted in no observed changes in cell attachment phenotypes. Phase contrast images for MSC, MG63 and HaCaT cells across 24 h, 48 h and 72 h post-exposure. Labels of no cell attachment denote scenarios in which cells could not adhere to the growth substrate post-exposure and thus could not be assessed. Scale bar, 50 µm.

Table S 1. Quantification of 24 h live cell percentage calculated from live/dead fluorescence staining, with data presented as mean ± SD from triplicate samples passed through the 10 µL min^{-1}; 25 µm high channel.

	MSC [%]	MG63 [%]	L929 [%]	HaCaT [%]
**TCP Ctrl**	96.9 ± 2	97.0 ± 4	96.5 ± 3	95.7 ± 2
**Flow Ctrl**	93.6 ± 3	97.7 ± 2	97.3 ± 2	96.9 ± 1
**400 mV**	-	96.3 ± 3	97.6 ± 2	96.7 ± 2
**800 mV**	-	-	96.3 ± 3	93.7 ± 4

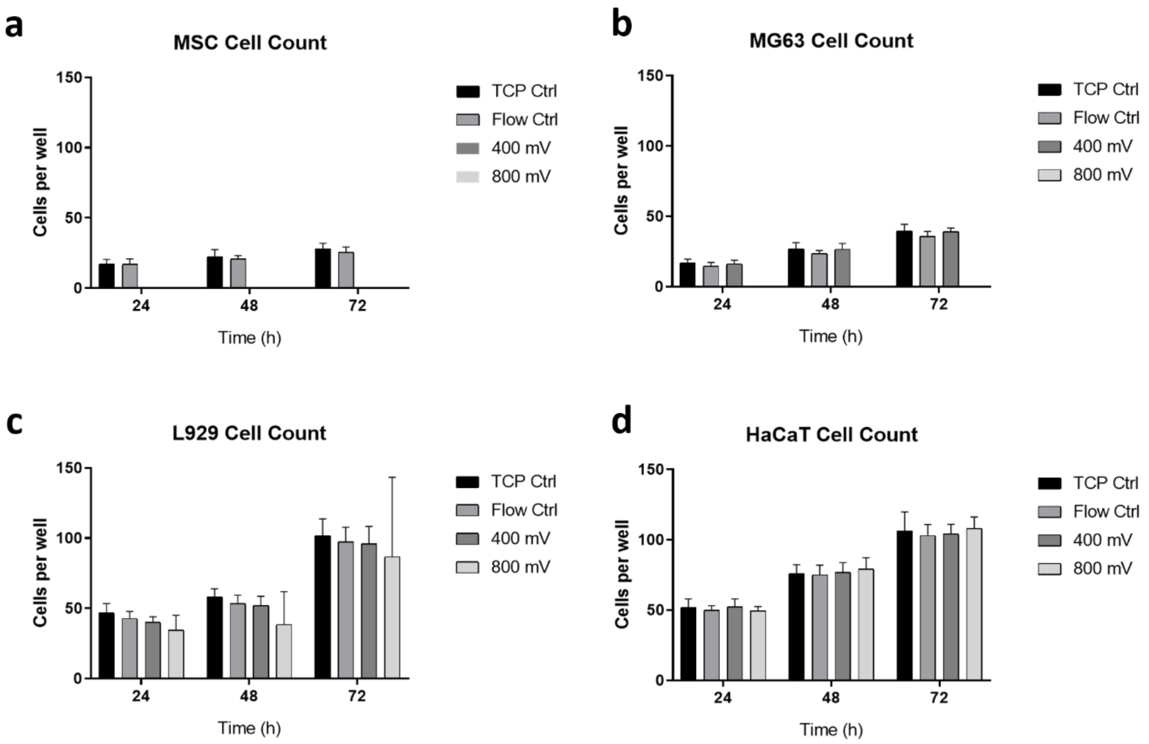

Figure S 4. Cell count data at three time points for MSCs (a), MG63 (b), L929 (c) and HaCaT (d).

Table S 2. Quantification of 24 h live cell percentage calculated from live/dead fluorescence staining, with data presented as mean ± SD from triplicate samples passed through the 10 µL min^{-1}; 50 µm high channel.

	MSC [%]	MG63 [%]
**TCP Ctrl**	97.1 ± 4	97.7 ± 2
**Flow Ctrl**	95.9 ± 3	96.4 ± 4
**400 mV**	95.3 ± 5	97.3 ± 3
**800 mV**	7.03 ± 6	95.6 ± 5

Table S 3. Quantification of 24 h live cell percentage calculated from live/dead fluorescence staining, with data presented as mean ± SD from triplicate samples passed through the 5 µL min^{-1}; 25 µm high channel.

	L929 [%]	HaCaT [%]
**TCP Ctrl**	97.7 ± 3	95.7 ± 4
**Flow Ctrl**	96.7 ± 3	95.3 ± 2
**400 mV**	96.2 ± 4	93.7 ± 3
**800 mV**	-	94.2 ± 4

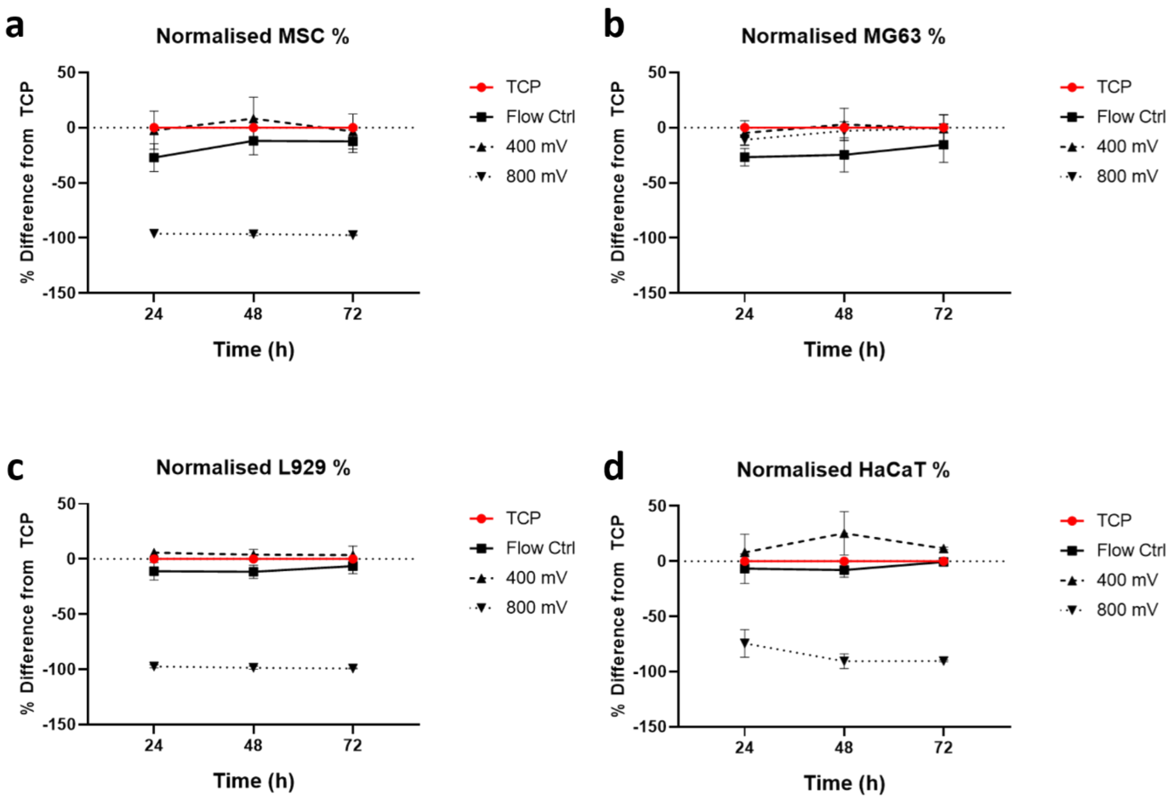

Figure S 5. Percentage difference in cell metabolic activity normalised to TCP for MSC (a), MG63 (b) based on results reported in Figure 6a and b (at 10 µL min^{-1} in a 50 µm channel height) and for L929 (c) and HaCaT (d) based on results reported in Figure 6c and d (at 5 µL min^{-1} in a 25 µm channel height).

Table S 4. Normalised range of metabolic activity highlighting the decreasing trend in range as cell stiffness increases (as shown in Figure 8a) based on results reported in (Figure S5) at a 400 mV acoustic exposure at 72 h.

	MSC [%]	MG63 [%]	L929 [%]	HaCaT [%]
**Min**	-18.18	-15.45	-1.49	10.19
**Max**	13.37	7.13	13.04	12.50
**Range**	31.55	22.57	14.53	2.31
